# Evolution of public hospitals expenditure by healthcare area in the Spanish National Health System: the determinants to pay attention to

**DOI:** 10.1186/s12913-018-3445-7

**Published:** 2018-09-10

**Authors:** Manuel Ridao-López, Micaela Comendeiro-Maaløe, Natalia Martínez-Lizaga, Enrique Bernal-Delgado

**Affiliations:** 1 Health Services and Policy Research Group (ARiSHP), Instituto Aragonés de Ciencias de la Salud (IACS), Zaragoza, Spain; 2 Red de Investigación en Servicios de Salud en Enfermedades Crónicas (REDISSEC), Madrid, Spain

**Keywords:** Hospital economics, Public hospitals, Public services, Public hospitals expenditure, Hospital services utilisation, Healthcare area expenditure variability, I18H42H51

## Abstract

**Background:**

In Spain, hospital expenditure represents the biggest share of overall public healthcare expenditure, the most important welfare system directly run by the Autonomous Communities (ACs). Since 2001, public healthcare expenditure has increased well above the GDP growth, and public hospital expenditure increased at an even faster rate. This paper aims at assessing the evolution of need-adjusted public hospital expenditure at healthcare area level (HCA) and its association with utilisation and ‘price’ factors, identifying the relative contribution of ACs, as the main locus of health policy decisions.

**Methods:**

Ecological study on public hospital expenditure incurred in 198 (HCAs) in 16 Spanish ACs, between 2003 and 2015. Aggregated and annual log-log multilevel models, considering ACs as a cluster, were modelled using administrative data. HCA expenditure was analysed according to differences in population need, utilization and price factors. Standardised coefficients were also estimated, as well as the variance partition coefficients.

**Results:**

Between 2003 and 2015, over 59 million hospital episodes were produced in Spain for an overall expenditure of €384,200 million. Need-adjusted public hospital expenditure, at HCA level, was mainly associated to medical and surgical hospitalizations (standardized coefficients 0.32 and 0.28, respectively). The ACs explained 42% of the variance not explained by HCA utilization and ‘price’ factors.

**Conclusions:**

Utilization, rather than ‘price’ factors, may be explaining the difference in need-adjusted public hospital expenditure at HCA level in Spain. ACs, third-payers in the fully devolved Spanish National Health System, are responsible for a great deal of the variation in hospital expenditure.

**Electronic supplementary material:**

The online version of this article (10.1186/s12913-018-3445-7) contains supplementary material, which is available to authorized users.

## Background

Healthcare is, in terms of government expenditure, the most important welfare system directly run by the Autonomous Communities (ACs) in Spain; in 2015 it represented between 3.9 and 9.5% of the regional GDP. Hospital expenditure is by far the biggest share, accounting for 62.4% of the overall public healthcare expenditure in 2015 [1]. Since 2001, when the healthcare devolution process to the ACs was fully completed [2], public expenditure in healthcare has increased, in aggregated terms, well above the GDP growth (85.1 vs. 54.4%); public hospital expenditure increased at an even faster rate (120%).

**Fig. 1:**
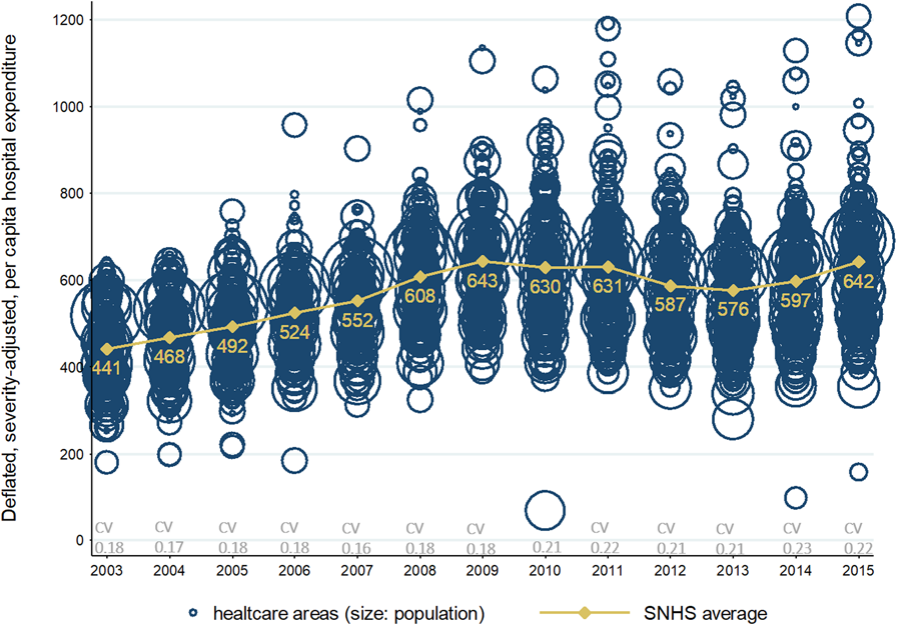
Evolution of deflated, severity adjusted, per-capita hospital expenditure by healthcare area. Bubble size corresponds to healthcare area size, in terms of inhabitants. Orange line shows Spanish National Health System average hospital, severity-adjusted, per capita expenditure along the period (in constant euros, base year 2003). CV, at the bottom, stands for the coefficient of variation

Since 2009, when the economic and financial crisis put at stakes the viability of the Spanish National Health System (SNHS) funding mechanisms (tax revenues plummeted), the Stability Program for the Kingdom of Spain [3] established cost-containment policies that translated into a significant decrease in public healthcare expenditure - 12% by 2013 [4]. Interestingly, this reduction was noticeably uneven across ACs (locus where financing and policy-making decisions are taken) and healthcare areas (HCAs) (locus where hospital and primary care services are provided). Figure 1 shows the downturn in deflated per capita severe-adjusted hospital expenditure after 2009 and later recovery from 2014. But most importantly, it also highlights the increase of hospital per capita expenditure variation across HCAs.

The underlying drivers of public hospital expenditure at HCA level have never been formally studied in the SNHS. Using routine hospital administrative data, this paper aims at exploring the association of various factors and hospital expenditure at HCA level care, as well as estimating the relative importance of ACs in the differences in expenditure.

## Methods

### Design and population

We carried out an observational ecological study on public hospital expenditure incurred in 198 HCAs in 16 Spanish ACs between 2003 and 2015. The sample accounted for 98.9% of the overall hospital admissions produced in the country in the period (59 million discharges).

### Variables

The main endpoint was need-adjusted hospital expenditure at HCA level. Instrumentally, the unitary price for each admission was allocated to the patient’s HCA of residence using *geo* codes (zip codes or equivalents, depending on the AC) and following the methodology developed in the Atlas of Variations in Medical Practice for the Spanish National Health System (Atlas VPM) [5, 6]. This unitary price per admission differed from hospitals and was obtained dividing the official consolidated hospital expenditure (excluding investments) [7] by the total amount of admissions at each hospital in a defined year. The hospital expenditure in each healthcare area was the result of the aggregation of those admission costs in the area, deflated at constant euros, base year 2003.

Independent variables: HCA expenditure was analysed according to differences in population need, utilization and price factors.

*Need* was defined using four different ecological variables: a) *human sex ratio* as the ratio of males to females residing in a HCA; b) *ageing index* as the ratio of people aged 65 and older to individuals aged 15 and younger; c) *over-ageing index* as the proportion of people aged 75 and older to those aged 64 and older; and d) *burden of disease* (i.e., population morbidity) as the cumulative number of hospitalisations for hip fracture, acute myocardial infarction, ischaemic stroke and cancer of the colon, lung or breast treated surgically aged 40 and older. These hospitalisations very likely reflect differences in health populations across HCAs and not differences in supply-side factors [8, 9].

*Utilization* was defined as the overall activity produced in the HCA in the period of study, differentiating between *medical hospitalizations*, *surgical admissions* and *outpatient day-case surgeries* (DC).

Finally, *price factors* (as the Spanish National System is not market-based and is highly regulated, prices and costs might be considered as equivalents) were approached according to three subsets of factors: *relative price of a stay*, *length of stay* and *structural features*; thus: a) relative price of a stay was constructed using APR-DRGs (All Patient Refined Diagnostic Related Groups) weights. Operationally, the overall price for a HCA was calculated as the ratio between the weighted sum of the total number of discharges and the total amount of discharges; the variable was split into medical and surgical admissions; b) length of stay (ALOS) in the HCA, expressed in days, distinguishing between medical and surgical admissions; and c) structural costs; as: i) whether a HCA is equipped with a tertiary referral hospital (namely, *Tertiary*) which requires a hemodynamic unit, a linear accelerator and more than 500 beds; ii) *medical staff workforce*, as the ratio of medical professionals to beds in the HCA; iii) *nursing staff workforce*, as the ratio of nurses to beds in the HCA; and, iv) *teaching hospital capacity*, as the ratio of medical residents to total medical professionals in the HCA.

Lastly, as the HCAs are hierarchically embedded into ACs, the latter were included in the model to capture overall AC-level effect (e.g. differences in health care policies, differences in management by providers, differences in socioeconomic status of the population) that could have a differential effect on hospital expenditure.

### Analyses

Since hospital healthcare expenditure comes from multiplying utilization (‘q’) and prices (‘p’), log-log multilevel models of random effects with ACs as a cluster, were specified. All regressions included *need* in order to discard legitimate sources of variations in expenditure. Partial coefficients were estimated for each year in the series, and for the whole period. Moreover, standardized beta coefficients were obtained to avoid scale effects among the variables and ease the interpretation of covariates relative effect over time. Additionally, the variance partition coefficient (VPC) was obtained as a measure of the cluster’s general effect, equivalent to the average effect of latent unobserved variables at AC level. If the effect was not relevant in the explanation of the variation in HCA expenditure, the VPC value would equal zero. All the models were assessed using likelihood ratio tests and adjusted coefficient of determination. Mathematical specifications were coded and analysed using Stata v.14. (see Additional file 1).

### Sources of information

Three data sources were used: 1) population demographics were drawn from the Municipal Censuses, gathered by the Spanish National Institute of Statistics (INE) [10]; 2) burden of disease, hospitalizations, day case surgery, relative prices and length of stay were obtained from the hospital administrative data infrastructure maintained by the Atlas VPM project [4]; in particular, relative prices were obtained using the APR-DRGs grouper licensed to the Atlas VPM group by 3 M; 3) data on workforce and overall hospital budget came from the Annual Hospital Survey (in Spanish*, Estadistica de Establecimientos Sanitarios en Regimen de Internado*).

## Results

Between 2003 and 2015 (Table 1), over 59 million hospital episodes were produced in Spain for an overall expenditure of €384,157 million (€325,967 million in constant terms). Referred to 2003, figures in 2015 represented an 95.2% relative increase, €17,516 million growth (€10,081 million in constant euros). Noticeably, there was a trend change in 2009 when expenditure reached its highest annual amount, €28,812 million, followed by a decline, reaching a new minimum in 2013 (€25,475 million) recovering again, in 2015, getting close to highest expenditure figures, €28,485 million.

**Table 1:** Descriptive figures throughout the period of analysis

		2003	2004	2005	2006	2007	2008	2009	2010	2011	2012	2013	2014	2015
SPENDING	HCA hospital expenditure (million €)	18,404	20,528	22,847	24,965	27,836	31,391	33,739	33,857	34,933	33,568	32,810	33,359	35,920
	Deflated HCA hospital expenditure (million €, base year 2003)	18,404	19,892	21,333	22,737	24,290	27,015	28,812	28,074	28,286	26,410	25,754	26,475	28,485
	HCA per capita hospital expenditure (€)	431.77	457.62	481.25	512.04	539.64	594.58	628.27	616.17	617.14	573.34	563.09	583.29	626.76
NEED	Masculinity index (base 100)	98.73	98.82	99.23	99.44	99.42	99.70	99.41	99.29	99.08	98.90	98.49	98.30	98.10
	Ageing index (base 100)	138.39	137.48	135.85	136.28	135.10	133.63	133.16	133.03	134.25	135.32	136.51	138.80	141.46
	Over-ageing (proportion)	44.95	46.16	47.54	48.24	49.30	50.39	51.12	51.50	51.85	52.36	52.29	51.46	50.33
	Burden of disease (average counts)	773.81	779.36	795.31	813.11	831.11	854.22	873.11	863.60	861.60	876.83	889.92	885.47	902.70
UTILIZATION	Hospital discharges (million)	4.01	4.14	4.25	4.37	4.54	4.62	4.68	4.75	4.78	4.77	4.87	4.97	5.14
	Outpatient case-day surgery (million)	0.64 (CV:1.04)	0.73 (CV:0.99)	0.80 (CV:0.97)	0,87 (CV:0.94)	0,91 (CV:0.94)	0,99 (CV:0.94)	1,05 (CV:0.94)	1,10 (CV:0.92)	1,18 (CV:0.89)	1,21 (CV:0.89)	1,32 (CV:0.89)	1,39 (CV:0.92)	1,49 (CV:0.93)
	Medical hospitalisations (million)	2.16 (CV:0.75)	2,18 (CV:0.75)	2,22 (CV:0.74)	2,24 (CV:0.74)	2,30 (CV:0.76)	2,34 (CV:0.74)	2,32 (CV:0.75)	2,26 (CV:0.74)	2,27 (CV:0.73)	2,26 (CV:0.75)	2,23 (CV:0.76)	2,26 (CV:0.75)	2,31 (CV:0.76)
	Surgical hospitalisations (million)	1.19 (CV:0.80)	1,22 (CV:0.80)	1,22 (CV:0.80)	1,25 (CV:0.79)	1,27 (CV:0.79)	1,30 (CV:0.80)	1,32 (CV:0.80)	1,32 (CV:0.80)	1,29 (CV:078)	1,28 (CV:0.79)	1,31 (CV:0.78)	1,31 (CV:0.79)	1,33 (CV:0.81)
PRICE	Relative price for a medical stay	6671 (CV:0.79)	6786 (CV:0.79)	6963 (CV:0.78)	7039 (CV:0.77)	7338 (CV:0.79)	7573 (CV:0.78)	7637 (CV:0.79)	7608 (CV:0.78)	7694 (CV:0.76)	7765 (CV:0.78)	7761 (CV:0.79)	7895 (CV:0.79)	8174 (CV:0.84)
	Relative price for a surgical stay	7004 (CV:0.85)	7242 (CV:0.85)	7345 (CV:0.85)	7615 (CV:0.84)	7820 (CV:0.84)	8038 (CV:0.85)	8309 (CV:0.84)	8409 (CV:0.85)	8394 (CV:0.82)	8448 (CV:0.83)	8714 (CV:0.82)	8847 (CV:0.83)	8983 (CV:0.85)
	Average length of stay - medical hospitalisation	6.96 (CV:0.14)	6.95 (CV:0.14)	6.99 (CV:0.15)	6.83 (CV:0.14)	6.89 (CV:0.15)	6.84 (CV:0.14)	6.94 (CV:0.19)	6.75 (CV:0.16)	6.60 (CV:0.14)	6.55 (CV:0.14)	6.55 (CV:0.13)	6.54 (CV:0.13)	6.74 (CV:0.15)
	Average length of stay - surgical hospitalisation	7.65 (CV:0.18)	7.54 (CV:0.18)	7.55 (CV:0.17)	7.46 (CV:0.17)	7.39 (CV:0.17)	7.34 (CV:0.16)	7.33 (CV:0.21)	6.95 (CV:0.18)	6.83 (CV:0.16)	6.80 (CV:0.16)	6.65 (CV:0.15)	6.54 (CV:0.14)	6.53 (CV:0.15)
	Tertiary (percentage of areas)	30.3	30.3	30.3	30.3	30.3	30.3	30.3	30.3	30.3	30.3	30.3	30.3	30.3
	Ratio medical staff to functioning beds	0.58 (CV:0.18)	0.61 (CV:0.19)	0.63 (CV:0.18)	0.64 (CV:0.21)	0.67 (CV:0.20)	0.71 (CV:0.19)	0.73 (CV:0.19)	0.75 (CV:0.22)	0.76 (CV:0.20)	0.76 (CV:0.22)	0.75 (CV:0.21)	0.75 (CV:0.23)	0.78 (CV:0.20)
	Ratio nursing staff to functioning beds	1.05 (CV:0.22)	1.09 (CV:0.22)	1.11 (CV:0.22)	1.13 (CV:0.24)	1.16 (CV:0.23)	1.22 (CV:0.20)	1.24 (CV:0.19)	1.19 (CV:0.21)	1.20 (CV:0.21)	1.18 (CV:0.20)	1.17 (CV:0.20)	1.19 (CV:0.21)	1.21 (CV:0.21)
	Ratio medical staff in training (MIR) to total medical professionals	0.21 (CV:0.62)	0.21 (CV:0.60)	0.21 (CV:0.60)	0.22 (CV:0.74)	0.20 (CV:0.61)	0.21 (CV:0.57)	0.20 (CV:0.55)	0.23 (CV:0.52)	0.24 (CV:0.49)	0.25 (CV:0.48)	0.26 (CV:0.48)	0.26 (CV:0.49)	0.23 (CV:0.50)

Comparing the evolution of need factors between 2015 and 2003, the population increased 9.2% (46 million inhabitants), the *human sex ratio* slightly increased until 2008 equalling the number of men to women, decreasing from there on to its lowest level in 2015 with 2% less men than women. The *ageing index* slightly decreased, reaching its minimum in 2010 (1.33) to increase afterwards at a higher level (1.42) so that there were in 2015 41.5% more inhabitants older than 65 than youngsters under 15. *The over ageing index or* percentage of very elderly grew, in aggregated terms, by 5.4 percentage points, although it also showed two stages, a first increase from 45 to 52.4 in 2012, decreasing again to 50.3 in 2015. The healthcare area’s average burden of disease constantly increased up to a 16.7% during the period of analysis. When it comes to utilization, overall, annually hospital activity increased by 28.2% (1.13 million episodes). Referring to 2003, the highest growth was observed in *day-case surgeries*, up to 131% (846,040 more procedures a year). *Medical and surgical admissions* represented, respectively, the 49 and 28% of hospital activity. Both showed similar evolution patterns during the period of analysis, identifying three stages, a first period of growth from 2003 to 2008–2009, increasing up to 8% (*medical admissions*) and 10.7% (*surgical admissions*), a second period of decrease up to 4.8 and 2.5% respectively, followed, from 2013 to 2012 on, by a recovery, up to levels prior to the application of cost containment measures, increasing a 3.9% (medical) and a 3.1% (surgical). In the case of ‘prices’, the annual *relative price of stay* increased by 22.5% in medical hospitalisations and 28.3% in surgical admissions, average *length of stay* slightly decreased (1.13 days in surgical stays, and 0.23 days in medical ones), and when it comes to structural features, while the condition of tertiary remained constant in the period, the *medical staff* ratio showed constant increase, in its aggregate 0.2 doctors per bed, *nursing staff* ratio showed two clear stages, an increasing constant trend from 2003 to 2009 (1.05 to 1.24 nurses per bed, respectively) to decrease until 1.17 in 2013 to recover earlier figures from then on. *Medical residents’* ratio remained constant at the beginning, increased from 2009 to 2013 (from 0.20 to 0.26 per medical professional, respectively) and decreased to 0.23 in 2015.

Table 2 shows the results of the multilevel regression analyses. When looking at the coefficients in the panel model, need variables *human sex ratio* and *ageing index* showed “protective” association with hospital expenditure overtime. On average, a 1% increase in the ratio of males to females would decrease hospital expenditure in a 0.4%. In the same direction, a 1% increase in the ratio of people aged 65 and older to people under 15 showed even a smaller reduction of hospital expenditure (− 0.05%). Only the proportion of the very elderly people showed a positive association with HCA’s hospital expenditure; thus, a 1% increase in the proportion of people aged over 75 years would increase HCA’s hospital expenditure by 0.5%.

**Table 2:** Log-log specified multilevel regressions of deflated hospital expenditure (*p*-values in brackets)

	2003	2004	2005	2006	2007	2008	2009	2010	2011	2012	2013	2014	2015	Aggregate panel
Intercept (fixed structural costs)	9633.8 (0.000)	7834.7 (0.000)	9969.3 (0.000)	13,271.0 (0.000)	25,391.3 (0.000)	30,900.0 (0.000)	46,625.0 (0.000)	37,512.9 (0.000)	17,574.4 (0.000)	10,836.5 (0.000)	9387.4 (0.000)	92,843.1 (0.000)	15,636.9 (0.000)	9798.2 (0.000)
Masculinity index	0.57 (0.026)	0.58 (0.066)	0.53 (0.030)	0.56 (0.013)	0.61 (0.061)	0.56 (0.038)	0.52 (0.019)	0.63 (0.131)	0.63 (0.116)	0.61 (0.090)	0.77 (0.330)	0.26 (0.063)	0.54 (0.040)	0.56 (0.000)
Ageing index	0.95 (0.013)	0.95 (0.031)	0.96 (0.125)	0.92 (0.001)	0.94 (0.014)	0.94 (0.020)	0.97 (0.193)	0.93 (0.016)	0.91 (0.001)	0.94 (0.024)	0.99 (0.618)	0.93 (0.019)	0.96 (0.097)	0.96 (0.000)
Over-ageing index 2	1.81 (0.108)	2.11 (0.053)	1.67 (0.182)	2.41 (0.002)	0.81 (0.368)	1.15 (0.718)	0.91 (0.637)	1.24 (0.541)	2.26 (0.003)	2.37 (0.001)	1.65 (0.070)	2.20 (0.028)	1.93 (0.013)	1.96 (0.000)
Ln (morbidity)	0.04 (0.381)	0.02 (0.779)	0.01 (0.881)	0.12 (0.023)	0.07 (0.213)	0.07 (0.202)	0.07 (0.203)	0.13 (0.009)	0.22 (0.002)	0.11 (0.071)	−0.02 (0.783)	0.24 (0.046	0.10 (0.068)	0.07 (0.000)
Ln (outpatient case-day surgery)	0.05 (0.006)	0.06 (0.023)	0.01 (0.638)	−0.01 (0.563)	0.02 (0.251)	−0.01 (0.823)	− 0.01 (0.652)	0.01 (0.781)	− 0.03 (0.244)	0.00 (0.933)	0.02 (0.631)	0.01 (0.892)	−0.06 (0.033)	0.02 (0.018)
Ln (medical hospitalisations)	0.33 (0.000)	0.34 (0.000)	0.41 (0.000)	0.33 (0.000)	0.31 (0.000)	0.34 (0.000)	0.27 (0.000)	0.08 (0.260)	0.29 (0.000)	0.42 (0.000)	0.42 (0.000)	0.38 (0.000)	0.36 (0.000)	0.34 (0.000)
Ln (surgical hospitalisations)	0.46 (0.010)	0.42 (0.064)	0.35 (0.104)	0.25 (0.134)	0.19 (0.289)	0.14 (0.486)	0.15 (0.395)	0.77 (0.000)	0.62 (0.002)	0.47 (0.012)	0.37 (0.080)	0.30 (0.247)	0.50 (0.005)	0.30 (0.000)
Ln (surgical *price of stay*-complexity)	0.10 (0.553)	0.15 (0.487)	0.20 (0.339)	0.27 (0.095)	0.33 (0.067)	0.38 (0.061)	0.46 (0.008)	−0-07 (0.736)	− 0.16 (0.421)	−0.05 (0.805)	0.15 (0.468)	0.00 (0.988)	0.04 (0.836)	0.23 (0.000)
Ln (average length of stay - medical hospitalisations)	0.07 (0.448)	0.19 (0.088)	0.12 (0.327)	0.13 (0.179)	0.07 (0.495)	0.00 (0.970)	−0.07 (0.506)	−0.19 (0.093)	0.15 (0.200)	0.33 (0.002)	0.25 (0.025)	0.30 (0.014)	0.21 (0.015)	0.12 (0.001)
Ln (average length of stay - surgical hospitalisations)	0.29 (0.000)	0.21 (0.021)	0.23 (0.023)	0.23 (0.008)	0.31 (0.001)	0.29 (0.007)	0.21 (0.026)	0.51 (0.000)	0.34 (0.002)	0.17 (0.101)	0.13 (0.251)	−0.09 (0.571)	0.30 (0.003)	0.20 (0.000)
Tertiary	1.07 (0.001)	1.08 (0.003)	1.06 (0.039)	1.08 (0.000)	1.09 (0.000)	1.07 (0.005)	1.06 (0.023)	1.06 (0.031)	1.05 (0.049)	1.07 (0.006)	1.06 (0.015)	1.04 (0.284)	1.03 (0.200)	1.06 (0.000)
Medical staff/functioning beds	0.93 (0.541)	0.87 (0.236)	0.92 (0.498)	1.15 (0.118)	1.02 (0.852)	0.99 (0.878)	0.88 (0.186)	1.14 (0.166)	1.32 (0.009)	1.33 (0.002)	1.27 (0.032)	1.04 (0.786)	1.21 (0.040)	1.11 (0.000)
Nursing staff/functioning beds	1.19 (0.024)	1.39 (0.000)	1.38 (0.000)	1.12 (0.039)	1.21 (0.002)	1.22 (0.003)	1.30 (0.000)	1.18 (0.014)	1.21 (0.006)	1.08 (0.248)	1.05 (0.537)	1.25 (0.032)	1.12 (0.049)	1.14 (0.000)
Medical professionals in training/medical professionals	1.22 (0.014)	1.11 (0.341)	1.21 (0.094)	1.24 (0.000)	1.19 (0.084)	1.25 (0.059)	1.27 (0.046)	1.18 (0.202)	1.38 (0.007)	1.38 (0.003)	1.52 (0.000)	1.38 (0.018)	1.62 (0.000)	1.29 (0.000)
VPC (Regions) Confidence interval 95%	0.40 (0.20 0.63)	0.28 (0.10 0.54)	0.18 (0.04 0.46)	0.48 (0.26 0.71)	0.26 (0.08 0.55)	0.33 (0.13 0.60)	0.45 (0.23 0.69)	0.28 (0.10 0.56)	0.48 (0.26 0.70)	0.53 (0.31 0.74)	0.51 (0.29 0.72)	0.41 (0.24 0.68)	0.35 (0.15 0.61)	0.42 (0.26 0.60)
Likelihood-ratio test of sigma(u) = 0	25.35 (0.000)	12.84 (0.000)	5.41 (0.000)	25.43 (0.000)	9.84 (0.001)	17.58 (0.000)	28.10 (0.000)	11.59 (0.000)	31.23 (0.000)	29.58 (0.000)	33.42 (0.000)	26.11 (0.000)	14.32 (0.000)	531.24 (0.000)
Pseudo-adjusted R^2^	0.980	0.977	0.978	0.977	0.978	0.972	0.968	0.968	0.964	0.964	0.962	0.943	0.976	0.965

Partial beta coefficients in the Ln covariates can be directly interpreted as elasticities, measuring the relative change of health expenditure to variations in covariates as follows: a 1% variation in the Ln-covariate turns into a x% increase (decrease) in expenditure]; Beta coefficients in non-Ln covariates (Tertiary, Medical staff, Nursing staff and Medical professionals in training) have to be interpreted as follows: a 1 point increase (decrease) in the covariate turns into a x% increase (decrease) in expenditure. X equals the value of the coefficient

Once need was adjusted, utilization factors were observed consistently associated over the period, with the highest magnitude of association in the case of *medical admissions*; on average, a 1% increase in *medical admissions* turned into a 0.34% increase in hospital expenditure, while in *surgical hospitalisations* a 1% increase translated into a 0.3% expenditure growth. *Day-case surgery*, with an uneven behaviour over the years, showed the smallest association on average – a 1% increase in day-case surgeries represented a 0.02% expenditure growth.

In turn, ‘price’ factors also behaved unevenly. The *relative price of a stay* showed to be the third factor more associated with hospital expenditure, but only for surgical admissions; on average a 1% increase in that covariate turned into a 0.23% increase in expenditure. Annually, only those years were surgical admissions were not significant, the relative price for a stay of surgical admissions showed to be highly associated with hospital expenditure. On the other hand, the *length of stay* in surgical admission was found highly associated with hospital expenditure most of the years; on average, a 1% increase in length of stay represented a 0.20% growth in hospital expenditure. Nevertheless, annually speaking, length of stay in medical admission was unevenly found associated with hospital expenditure; on average, a 1% increase in the covariate turned into a 0.12% increase in expenditure. With regard to the structural costs, a consistent association was found over time in *tertiary*, *nursing staff* and *teaching capacity*, not however in *medical staff*; thus, the average expenditure increased by 6% in health care areas equipped with a tertiary hospital, 14% for each point of increase in nursing staff, and rough 29% for each point of increase in medical residents.

**Fig. 2:**
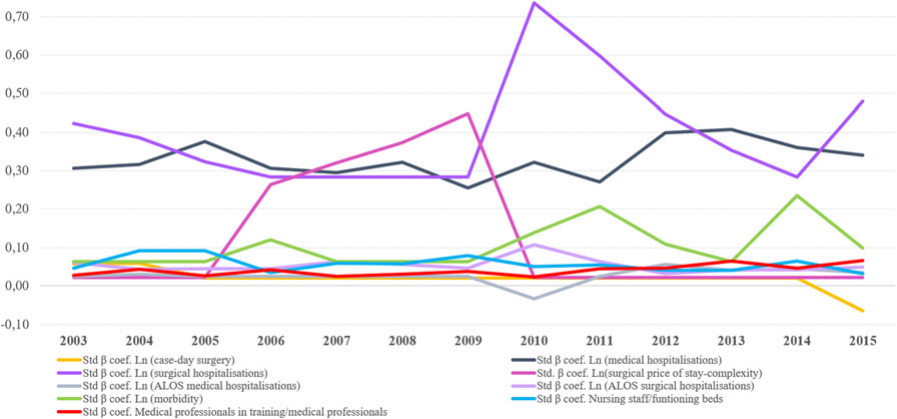
Standardised beta coefficients along the period

Standardized beta coefficients in Fig. 2 exhibit the actual impact of the different covariates of the study. Once scale effects were discarded, HCA hospital expenditure mainly lay in the variation in utilization, basically in hospital admissions, surgical and, above all, medical hospitalizations (overall standardized beta coefficients 0.28 and 0.32, respectively).

**Fig. 3:**
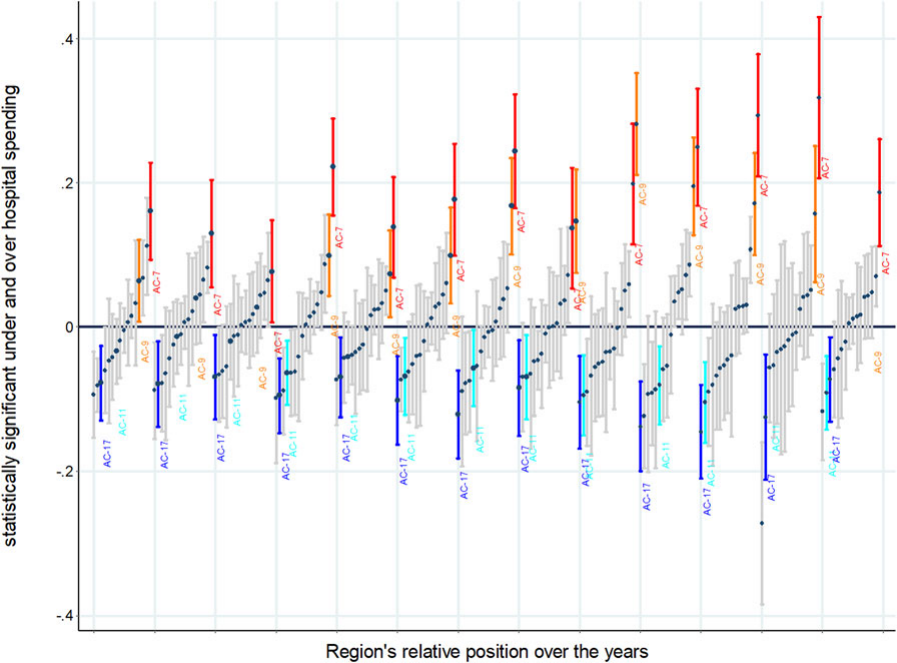
Region’s relative position according to their hospital spending along the period. Spikes are representing ACs expenditure residual (central estimation and 95% confidence interval) by year. The Central line represents the average HCA hospital expenditure in the 16 ACs. Those ACs above the line are expending statistically more than the average; ACs below the line are expending statistically less than the average. Some ACs are consistently above and some consistently below

Finally, the ACs-level unobserved factors explained a wealth of variance (42%) beyond the variance explained by the aforementioned HCA factors, ranging from 18% in 2005 to 53% of the residual variance in 2012 (Fig. 3).

## Discussion

In this ecological study on routine data, comparing HCA and ACs in the SNHS, differences in hospital utilization, to a lesser extent ‘prices’, and latent contextual factors at AC level have been found to be associated with need-adjusted HCA hospital expenditure.

### Utilisation vs. ‘price’

There is abundant literature evidencing the relatively higher importance of utilization factors (e.g. propensity to hospitalize, induced demand, etc.) when explaining healthcare expenditure [11–14]. At the same time, recent evidence [15–18] has found the opposite, giving price factors a more important role when explaining population’s healthcare expenditure. Likewise, in the Spanish literature, ecological studies have pointed to hospital utilisation as the main associated factor [19]. Our work, based on the largest data series ever published on this topic in Spain, is unequivocally pointing to utilization as the main associated factor.

This apparent inconsistency in comparison with international literature may reside in the fact that ‘prices’ in a non-market oriented and highly regulated system are expected not to vary across HCAs, nor over time. Indeed, utilization factors exhibited greater variation than all except one of the ‘price’ factors that showed consistent significance along the period.

### Why do medical hospitalisations explain more than surgical hospitalisations?

The relatively stronger association between medical hospitalisations (as compared with surgical admissions) and need-adjusted HCAs hospital expenditure may have several explanations. On the one hand, surgical ALOS has experienced a larger reduction in the period of analysis (from 7.65 to 6.53 days); this phenomenon, together with the increase in outpatient day-case surgery while keeping the same supply of beds, directly translated into more admissions. Medical ALOS, on the other hand, did not vary a similar extent (from 6.96 to 6.74 days), basically because medical departments do not have an alternative outpatient resource for early-discharge.

### The effect of autonomous communities

Finally, it is important to highlight the huge impact of ACs on need-adjusted HCA hospital expenditure. The high VPC values (Table 1) strongly suggest that unobserved factors at AC level are affecting HCAs differently.

It is very likely that unobserved factors between 2003 and 2009, when the growth in hospital expenditure was patent, will differ from those in the recession period and later recovery. When it comes to the former, just after the devolution process, ACs became third party payers whose budget is decided in the regional parliaments, and gained full responsibility for planning and service management; the uneven deployment of the devolution process across ACs might be behind the high VPC values. In turn, during the recession and onwards, we could hypothesize that the unequal AC reaction to the austerity measures are behind the ACs strong association with hospital expenditure; depending on the AC, the budgetary constraints ranged in public healthcare expenditure from a 4.4% reduction to a 31.3% reduction [1, 20–23]. In 2013, public healthcare expenditure began a recovery process, reaching in 2015 similar expenditure levels than in 2009, emphasising ACs’ variability, ranging the increases from 2.3 to 17.6% regarding 2013 figs. [1].

### Implications

According to the partial coefficients, all the actionable covariates (i.e. utilization and ‘price’ factors) are observed to be inelastic. Therefore, any action meant to reduce any of the factors by 1%, will have an impact on HCA hospital expenditure which is less than 1%. Translating average partial coefficients (panel model) into current euros highlights the potential impact of focusing on those factors found to be associated to hospital expenditure. Thus, taking the 2015 expenditure figures (the latest in the series) and accounting just for those actionable factors, a 1% reduction in medical admissions would turn into a reduction of €122.13 million, a 1% reduction in surgical hospitalizations would translate into a €107.8 million reduction, a 1% reduction in the relative price of a surgical admission would reduce expenditure by €82.6 million a year, a 1% length of stay reduction in surgical admissions would translate into an €71.8 million reduction, and a 1% length of stay reduction in medical admission would reduce expenditure by €43.1 million a year. Finally, when it comes to structural costs, reducing nursing staff, medical staff and medical residents by 1% would turn in a reduction of hospital expenditure of €58.3 million, €27.7 million and €16.5 million, respectively.

These figures would orient policies towards utilization rather than containment of ‘prices’, when it comes to reducing HCA hospital expenditure. Interestingly, most of the policies implemented in the country have been oriented towards reducing prices and production costs (e.g. reducing length of stay), or more recently increasing productivity. Notably, most of the measures included in the Stability Program for the Kingdom of Spain were oriented towards dealing with ‘price’, and those oriented towards ‘utilization’ only marginally affected hospital utilization. Indeed, as highlighted in a recent Atlas of Variation in low-value procedures in Spain, neither the trend nor the variation in the use of this kind of procedures have been observed to change after 2012, when major cost-containment measures were issued [24].

### Limitations

This paper aims at eliciting those factors that, beyond differences in need, are behind HCA hospital expenditure. It might be argued that adjusting ‘need’ by age, sex and burden of disease have not been sufficient and part of the variation in hospital expenditure might still be attributed to differences in need factors not accounted for. Indeed, socioeconomic factors have not been included as these factors are not annually available in Spain at HCA level for the whole period of analysis. Previous research in our context found a very small effect of socioeconomic differences on hospital utilization [9, 25]. All in all, as geographic analyses act as a natural experiment built upon big areas (75% of the HCA in our study have more than 94,000 inhabitants), the distribution of latent or unobserved need factors not included in the regression models will reflect a reasonably homogeneous distribution across HCA.

On the other hand, as the study aims at analysing hospital expenditure at HCA level, a critical issue was the unbiased allocation of hospital expenditure to the HCAs. The methodology has been validated over the years for the Atlas Project of Variations in Medical Practice in the SNHS [26–31]. Overall, 98.2% of hospital discharges and their costs were successfully allocated to the area of residence.

Finally, another critical issue is the accounting method used to estimate the price of a stay. The absence of a homogenous and universal full-cost accounting system for Spanish public hospitals led us to *proxy* the relative price of a stay using APR-DRG weights. More than 99% of the episodes in the study were adequately grouped; yet, using APR-DRG weights might entail coding phenomena (e.g. different coding practices). In order to evaluate this potential bias, we compared our APR-DRG weights with full-costing data from a small sample of Spanish public hospitals included in the RECHOSP network [32]. Correlation figures were higher than 60%, which makes APR-DRG weights an acceptable proxy when accounting for the price of a stay in our context.

## Conclusions

Need-adjusted HCAs hospital expenditure mainly lay in hospital utilization and to a lesser extent on ‘price’ factors. The ACs of residence, as the locus of decisions on health care financing, planning and management, hold a great deal of the variation in HCA hospital expenditure. Despite the concern about the evolution of healthcare expenditure, a few years after containment measures adoption and consequent reaction of decrease, hospital expenditure returned to its 2009 maximum levels, a proof of healthcare system hysteresis.

## Additional file

Additional file 1: Methodology: Statistical specifications. (DOCX 17 kb)

## Abbreviations

AC: Autonomous community
ALOS: Average length of stay
APR-DRG: All patients refined diagnostic related group
Atlas VPM: Atlas of variation in medical practice for the spanish national health system
DC: Outpatient day-case surgery
GDP: Gross domestic product
HCA: Healthcare area
INE: Spanish National Institute of statistics (*Instituto Nacional de Estadística*)
RECHOSP: Spanish Network of Hospital Costs (*Red Española de Costes Hospitalarios*)
SNHS: Spanish National Health System
VPC: Variance partition coefficient
