# Supplementary material for: Evolution of public hospitals expenditure by healthcare area in the Spanish National Health System: the determinants to pay attention to
Source: BMC Health Serv Res. 2018 Sep 10;18:696. doi: 10.1186/s12913-018-3445-7 (PMC6131833; doi:10.1186/s12913-018-3445-7)
Supplement: Supplementary file 1 — Methodology: Statistical specifications. (DOCX 17 kb) [file 12913_2018_3445_MOESM1_ESM.docx]

**Additional file**

***Methodology: Statistical specifications***

Since hospital healthcare expenditure comes from multiplying quantity (use of services) and price factors (from the utilization of factors of production), "q" and "p", we specified log-log multilevel models of random effects, with regions (Autonomous Communities) as cluster. Two types of consecutive regressions were developed, the first one with *time* as fixed effect in an aggregated panel data set from which we selected those explanatory variables statically significant (model 1),

$Y_{ij}=\gamma_{00}+\sum_{n=1}^{N} \gamma_{n0}x_{nij}+\sum_{k=1}^{K} \gamma_{k0}q_{kij}+\sum_{h=1}^{H} \gamma_{h0}z_{hij}+\sum_{t=1}^{T} \gamma_{t}D_{t}+u_{oj}+\varepsilon_{ij}$ (1)

Where: $Y_{ij}$: logarithm of hospital public expenditure of healthcare i from region j

$x_{nij}$: N predictors which characterize the population under coverage at each healthcare area i from region j

$q_{kij}$: K predictors capturing the need/use of healthcare hospital services at each healthcare area i from region j (demand factors)

$z_{hij}$: H predictors capturing factors of production which approach prices/expenses of hospital healthcare services provided at healthcare area i from region j (supply factors “p”)

$D_{t}$: dichotomous variables, one for each year of the period

$u_{oj}iidN\left( 0,\sigma_{u}^{2} \right)$ (random effect of region j)

$\varepsilon_{ij}iidN\left( 0,\sigma_{\varepsilon}^{2} \right)$ (random effect of healthcare area i from region j)

to continue independently developing simultaneous annual regressions, one for each of the years included in our period of analysis, all of them with the same covariates selected from the first model (model 2)

${Y'}_{ijt}={\gamma'}_{00t}+\sum_{n=1}^{N} {\gamma'}_{n0t}x_{nijt}+\sum_{k=1}^{K} {\gamma'}_{k0t}q_{kijt}+\sum_{h=1}^{H} {\gamma'}_{h0t}z_{hijt}+{u'}_{ojt}+{\varepsilon'}_{ijt}$ (2)
